# The Special Treatment of Nervous Exhaustion

**Published:** 1873-09

**Authors:** 


### THE SPECIAL TREATMENT OF NERVOUS EXHAUSTION.

Dr. Routh, of London, sums up his teachings on this subject, in the London *Medical Press and Circular*, as follows: His recommendations are

1. Complete rest, and especially abstention from all occupation resembling that upon which the mind has been overworked. This is a rock upon which many split. A friend of mine, an obstetrical physician of great eminence and practice, thought to take his holiday in Ireland, but in doing so could not resist the temptation of visiting the Irish hospitals. His two months passed pleasantly enough, but not so the following winter. He came out with an œdematous eruption, and lost his nails, and continued ill till Swiss air and Swiss mountains, during his next holiday, restored him. It is important, however, to give a patient some occupation. To let a man who has been in harness many years degenerate at once into perfect idleness is most unwise. But the work supplied should be altogether different from his former occupation. Where a new fancy has been adopted, and it is not likely to injure the person, it is well to encourage it. It calls forth into action new powers of the brain, while the previously overwrought parts have time to recover. I know a case where the weak man took to writing poetry. It was a perfect phrenzy. Day after day he wrote wondrous effusions, remarkable both for their beauty and pathos. I encouraged him. He completely recovered in the year.

Such a plan of treatment is, after all, carrying out the principle so accurately laid down by my friend Dr. Richardson, when he states, that the brain "is an organ that can rest in parts where jaded, and work in parts that are not jaded, at one and the same time."

2. It is essential to insure tranquillity to the senses which especially give incorrect impressions, and only to put those objects before them which will tend to soothe the mind. Before that of sight, green fields are most conducive to rest. There is no color which, like green, is so soothing to the eye. The hearing must, likewise, be soothed ; noisy places avoided. Sea change, so often advantgeous by reason of the bromine inspired, is to be avoided if the waves be stormy or otherwise than in a state of calm. Smell and touch, likewise, are to be calmed ; quiet, sweet-scented, zephyr breezes are the best. A host of quieting, mesmeric influences may be produced thereby, just as sleep can be induced by looking fixedly at any object, or hearing a monotonous voice, or feeling a gentle breeze playing upon the face. Taste is gratified in like manner by simple, nutritious diet, but especially fish diet, and that fish diet, for the reasons before stated, of those varieties richest in phosphorus. The best internal medicines are iron, as improving the sanguineous plasma ; but phosphorus, whether as atlotropic phosphorus, or as the solvent phosph. med. ♏ x. to xv. ter die, or as phosphide of zinc, affords the dest chance of restoration.

